# GADD45α Induction by Nickel Negatively Regulates JNKs/p38 Activation via Promoting PP2Cα Expression

**DOI:** 10.1371/journal.pone.0057185

**Published:** 2013-03-11

**Authors:** Yonghui Yu, Jingxia Li, Yu Wan, Jianyi Lu, Jimin Gao, Chuanshu Huang

**Affiliations:** 1 Nelson Institute of Environmental Medicine, New York University School of Medicine, Tuxedo, New York, United States of America; 2 Oversea Laboratory, Center for Medical Research, Wuhan University, Wuhan, Hubei, China; 3 Zhejiang Provincial Key Laboratory for Technology & Application of Model Organisms, School of Life Sciences, Wenzhou Medical College, Wenzhou, Zhejiang, China; University of Pittsburgh Cancer Institute, United States of America

## Abstract

Growth arrest and DNA damage (GADD) 45α is a member of GADD inducible gene family, and is inducible in cell response to oxidative stress. GADD45α upregulation induces MKK4/JNK activation in some published experimental systems. However, we found here that the depletion of GADD45α (GADD45α−/−) in mouse embryonic fibroblasts (MEFs) resulted in an increase in the phosphorylation of MKK4/7, MKK3/6 and consequently specific up-regulated the activation of JNK/p38 and their downstream transcription factors, such as c-Jun and ATF2, in comparison to those in GADD45α+/+ MEFs cell following nickel exposure. This up-regulation of MKK-JNK/p38 pathway in GADD45α−/− cell could be rescued by the reconstitutional expression of HA-GADD45α in GADD45α−/− MEFs, GADD45α−/−(HA-GADD45α). Subsequent studies indicated that GADD45α deletion repressed expression of PP2Cα, the phosphotase of MKK3/6 and MKK4/7, whereas ectopic expression of HA-PP2Cα in GADD45α−/− cells attenuated activation of MKK3/6-p38 and MKK4/7-JNK pathways. Collectively, our results demonstrate a novel function and mechanism responsible for GADD45α regulation of MKK/MAPK pathway, further provides insight into understanding the big picture of GADD45α in the regulation of cellular responses to oxidative stress and environmental carcinogens.

## Introduction

Nickel is a well-established human carcinogen that widely distributes in soil and water, and the main routes of nickel uptake are inhalation, ingestion, and dermal penetration [1]. Exposure to high levels of nickel compound results in lung cancer and nasal cancer [2]. Since nickel has a weak effect on DNA damage and mutation, it is thought that nickel’s epigenetic effect and nickel-initiated activation of signaling pathways lead to activation of transcription factors and the expression of their downstream genes is the major mechanism responsible for its carcinogenic effects [3]. Reports from others and our laboratories show that nickel activates many transcription factors, including NFκB, NFAT, and HIF-1α in various experimental systems [3], [4]. Nickel has been reported to induce phosphorylation of JNK in A549 cell [5], or p38 and Erk in dendric cell [6]. Our published studies initially show that nickel exposure induces VEGF expression through PI-3K/Akt/HIF-1α-dependent pathway [7], and that JNK activation by nickel compounds is crucial for its stabilization of HIF-1α protein by modulation of Hsp90 acetylation and stability [3]. Thus, elucidating JNK regulation is significant in understanding of nickel responses.

JNK and p38 are two major members of the MAPK family and are essential for the activation of many transcription factors that play a role in the regulation of various normal cellular functions and the development of numerous types of cancer. Activated JNK is associated with HTLV-mediated tumorigenesis [8] and inhibition of JNK phosphorylation reduces tumor growth in mouse tumorigenic models [9]. JNK2 has been reported to promote formation of human glioblastoma [10], while suppression of JNK2 can repress growth and induce apoptosis of human cancer cells [11]. In JNK2 deleted mice, tumor formation in two-stage skin carcinogenic mouse model is markedly reduced in comparison to that in wild type mice [12]. p38 has also been found to be involved in oxidative responses. Clinical studies show that p38 activity in the tissue of non-small lung cancer is higher than that in matched non-neoplastic lung tissue [13]. Furthermore, it has been reported that p38 is involved in UVB-induced skin carcinogenesis [14], and is required for ovarian cancer cell survival [15]. Therefore, exploring the mechanisms underlying the activation of JNK/p38 is of significance for the understanding of oxidative stress responses.

The growth arrest and DNA damage 45 (GADD45) is a family that consists of GADD45α, GADD45β, and GADD45γ [16], [17]. GADD45α has been considered as tumor suppressor, and is inducible in response to stress agents, such as UV radiation and arsenite [18], [19]. Previous studies indicate that GADD45α upregulation mediates JNK and p38 activation [20], and subsequently increases phosphorylation of c-Jun and ATF2 [21], [22]. On the other hand, the spontaneous phosphorylation of p38 at Tyr323 is observed in resting T cells that have been isolated from gadd45α−/− mice [23]. Recent studies indicate that GADD45α function as either tumor suppressor or promoter is dependent on stimulation of oncogenic stress [24]. GADD45α can suppress Ras-driven breast tumorigenesis through increasing JNK-mediated cell apoptosis, whereas it also promotes breast cancer development via down-regulating MMP10 in GSK3β/β-catenin dependent manner [24]. In current study, we demonstrate that GADD45α inducible expression due to nickel exposure provides an inhibitory effect on activation of MKK/JNK/p38 pathway via promoting PP2Cα expression.

## Materials and Methods

### Cells and Reagents

Primary culture GADD45α+/+ and GADD45α−/− MEFs were generous gift from Dr. Victor Tron, Department of Pathology and Molecular Medicine, Queen’s University (Kingston, Ontario) [25]. GADD45α+/+ and GADD45α−/− MEFs were cultured by us for over 9 months for immortalization in DMEM containing 10% fetal bovine serum (FBS). HCT116 cells were cultured in McCoy’s 5A medium containing 10% FBS. Nickel chloride was bought from Sigma-Aldrich (St. Louis, MO, USA). Antibodies against GADD45α, MTK1/MEKK4, Fos B, Jun B and PP2Cα were purchased from Santa Cruz Biotechnology Inc (Santa Cruz, CA, USA). c-Jun, phosphor-c-Jun at Ser63, phosphor-c-Jun at Ser73, ATF2, phosphor-ATF2 at Thr71, JNK, phosphor-JNK at Thr183/Tyr185, p38, phosphor-p38 at Thr180/Tyr182, Erk, phosphor-Erk at Thr202/Tyr204, MKK4, phosphor-MKK4 at Ser257/Thr261, MKK7, phosphor-MKK7 at Ser271/Thr275, MKK3, phosphor-MKK3/6 at Ser189/207 were purchased from Cell Signaling Technology (Beverly, MA, USA). Antibodies against β-Actin and Flag were from Sigma-Aldrich (St. Louis, MO, USA) and HA antibody was bought from Covance (Princeton, NJ, USA). The antibody of phosphor-MTK1 at Thr1493 was made by Mutsuhiro Takekawa’s group as described in previous study [26].

### Constructs and Transfection

Expression vector pcDNA3/HA-GADD45a was described in previous published study [27], and pcDNAIamp/Flag-MTK1 was kindly provided by Dr. Mutsuhiro Takekawa [26]. Adenovirus with expression of HA-PP2Cα and its control vector were kindly provided by Dr. Shinri Tamura [28]. FuGENE 6 transfection reagent (Roche Applied Science, Indianapolis, IN, USA) was used to carry out the stable transfection according to the protocol instructions provided by manufacturer. pcDNA3/HA-GADD45α was stably transfected into GADD45α−/− cells [19] and HCT116 cells. AP-1 luciferase reporter was purchased from Stratagene (La Jolla, CA, USA) and was co-transfected with pSUPER vector into GADD45α+/+, GADD45α−/− and GADD45α−/−(HA-GADD45α) cells, respectively.

### Reverse Transcription PCR

Cells were treated with or without NiCl_2_, as indicated in figure legends. Total RNA was extracted using TRIZOL reagent (Invitrogen, Carlsbad, CA, USA) following the manufacturer’s instructions. SuperScript™ III First-Strand Synthesis System (Invitrogen, Carlsbad, CA, USA) was used to synthesize first-strand cDNA with oligo (dT)_20_. Specific primer pairs were designed to amplify *gadd45α* (Forward: 5′-ATGACTTTGGAGGAATTCTCG-3′, Reverse: 5′-CACTGATCCATGTAGCGACTT-3′), *pp2cα* (Forward: 5′-GGGTGAATTGGCAAGCAAGCGG-3′, Reverse 5′-GGCGAAGGTGAGTCACGGC-3′), and *β-actin* (Forward: 5′-CATCCGTAAAGACCCTCTATGCC-3′, Reverse: 5′-ACGCAGCTCAGTAACAGTCC- 3′).

### Real-time PCR

GADD45α+/+ (vector), GADD45α−/− (vector) and GADD45α−/−(HA-GADD45α) cells were seeded into 6-well plates, respectively. When cell confluence reached 70∼80%, 5 µM Actinomycin D, the inhibitor of RNA synthesis, was used to treat cells for indicated time periods, and total RNA samples were collected using TRIZOL reagent (Invitrogen, Carlsbad, CA, USA). First strand cDNA and real-time PCR were performed as previously described [29]. The primers of *pp2cα* (Forward: 5′-GGGTGAATTGGCAAGCAAGCGG-3′, Reverse 5′-GGCGAAGGTGAGTCACGGC-3′) and *β-actin* (Forward: 5′-CCTGTGGCATCCATGAAACT-3′, Reverse: 5′-GTGCTAGGAGCCAGAGCAGT-3′) were used for amplification.

### AP-1 Activity Assay

GADD45α+/+/AP-1-Luc, GADD45α−/−/AP-1-Luc and GADD45α−/−(HA-GADD45α)/AP-1-Luc transfectants were seeded into 96-well plate, respectively. After cell density reached 70∼80% confluence, the cell culture medium was replaced with 0.1% FBS DMEM and cultured for 12 hrs. The cells were then exposed to NiCl_2_, as indicated in figure legends. Luciferase activity was measured, and AP-1 activity was presented as a luciferase activity relative to medium control (Relative AP-1 activity), as described in previous study [30].

### Western Blot Assay

Cells were treated with NiCl_2_ for the indicated doses and time periods, and were extracted with boiling buffer (10 mM Tris–HCl, pH 7.4, 1% SDS, and 1 mM Na_3_VO_4_). Protein concentrations were determined by Nano Drop 1000 (Thermo Scientific, Holtsville, NY, USA). Extracted proteins (30–60 µg) were applied to Western blotting assay as described in our published study [30].

### Immunoprecipitation

For determination of GADD45α interaction with MTK1, HCT116 (vector) and HCT116 (HA-GADD45α) cells were cultured in 10-cm dishes till 70∼80% confluence. The cell culture medium was replaced with 0.1% FBS McCoy’s 5A and cultured for 12 hrs, and cells were then treated with NiCl_2_ for 3 hrs. For evaluation of GADD45α function in MTK1 auto-phosphorylation, Flag-MTK1 were transiently transfected into HCT116 (vector) and HCT116 (HA-GADD45α) cells. 24 hrs after the transfection, cells lyses were collected with IP buffer (25 mM Tris-HCl pH 7.5, 1 mM DTT, 30 mM MgCl_2_, 40 mM NaCl, 0.5% NP-40 and protease inhibitor). 2 mg total protein from each sample was used to incubate and rotate with antibodies specific against HA (anti-HA, Abcam) or Flag (anti-Flag, Sigma) together with protein A/G plus agarose (Santa Cruz, CA) overnight at 4°C. Agarose were collected after centrifugation (5000 g, 2 min) at 4°C and washed using IP buffer for 4∼5 times. 2×SDS sample buffer was used to prepare western blot samples.

### Statistical Methods

The Student’s t-test was utilized to determine the significant difference among GADD45α+/+, GADD45α−/− and GADD45α−/− (HA-GADD45α) cells upon NiCl_2_ exposure. The differences were considered to be significant at P≤0.05.

## Results

### GADD45α induction by Nickel Inhibited Activation of JNK and p38

**Figure 1:**
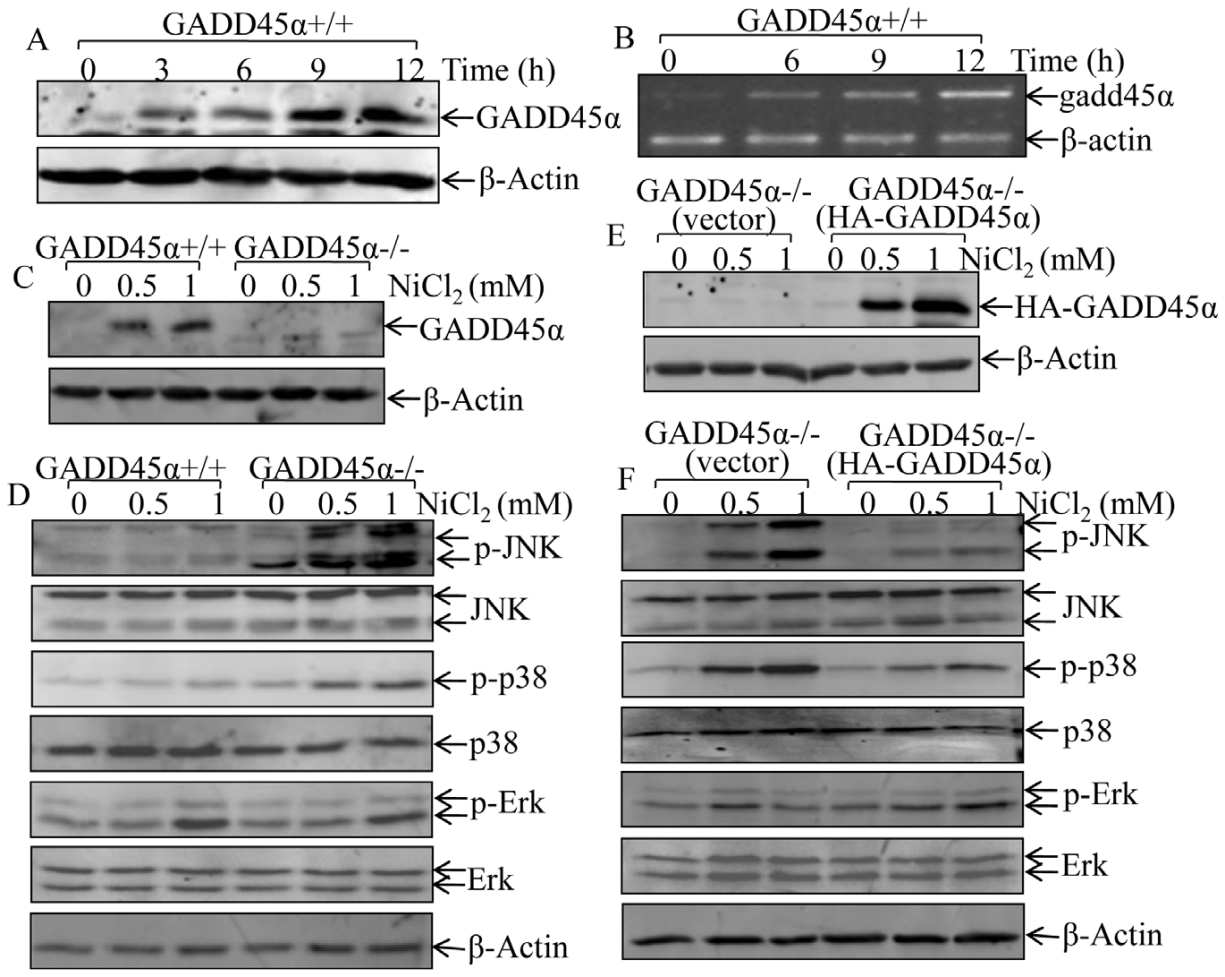
GADD45α induction mediated the inhibition of JNK and p38 activation due to nickel exposure. (A–F), GADD45α+/+ and GADD45α−/− cells were exposed to NiCl_2_ at doses and time periods as indicated. The cells were collected using cell lysis buffer (A, C–F) for protein extraction or TRIZOL (B) for mRNA extraction as described in the section of “Materials and Methods”. The GADD45α protein expression and MAPK activation were determined by Western blot (A, C–F) and gadd45α mRNA level was evaluated by RT-PCR (B).

GADD45α has been reported to be an inducible expression in response to oxidative stress, such as UV radiation [18] and arsenite exposure [19]. To determine the potential involvement of GADD45α in nickel responses, cells were treated with NiCl_2_ at 1 mM for various time periods. The results showed that nickel exposure induced GADD45α expression in both protein and mRNA levels in time-dependent manner (Figs. 1A and 1B). Our published studies demonstrate that up-regulation of GADD45α expression, by increasing its protein de-ubiquitination, is responsible for arsenite activation of the JNK-dependent apoptotic pathway [19]. In order to explore the role of GADD45α in regulating MAPKs activation due to nickel exposure, we established spontaneous immortalized GADD45α+/+ and GADD45α−/− MEF cells, which were identified in Fig. 1C. Furthermore, our results showed that deletion of GADD45α markedly enhanced activation of both JNK and p38 upon nickel exposure, whereas there was no observable alteration on Erk activation (Fig. 1D). To exclude the potential contribution of other gene mutations during the spontaneous immortalization process to upregulation of JNK/p38 activation with nickel treatment in GADD45α−/− MEFs, pcDNA3/HA-GADD45α expression construct was used to reconstitute GADD45α expression in GADD45α−/− cell. As shown in Fig. 1E, although the basal level of HA-GADD45α expression was very low due to its fast degradation in MEF cells, nickel treatment obviously stabilized HA-GADD45α protein. Moreover, reconstitutional expression of HA-GADD45α in GADD45α−/− MEFs (Fig. 1E) dramatically inhibited the activation of JNK/p38 following nickel exposure, while it had no marked effect on Erk activation (Fig. 1F). Our results indicate that nickel exposure induces GADD45α expression, and that this GADD45α induction provides an inhibitory effect on nickel-induced JNK/p38 activation, which might account for relatively weak effect of nickel on activation of JNK/p38/AP-1 pathway that has been reported in our previous studies in normal cells [4].

**Figure 2:**
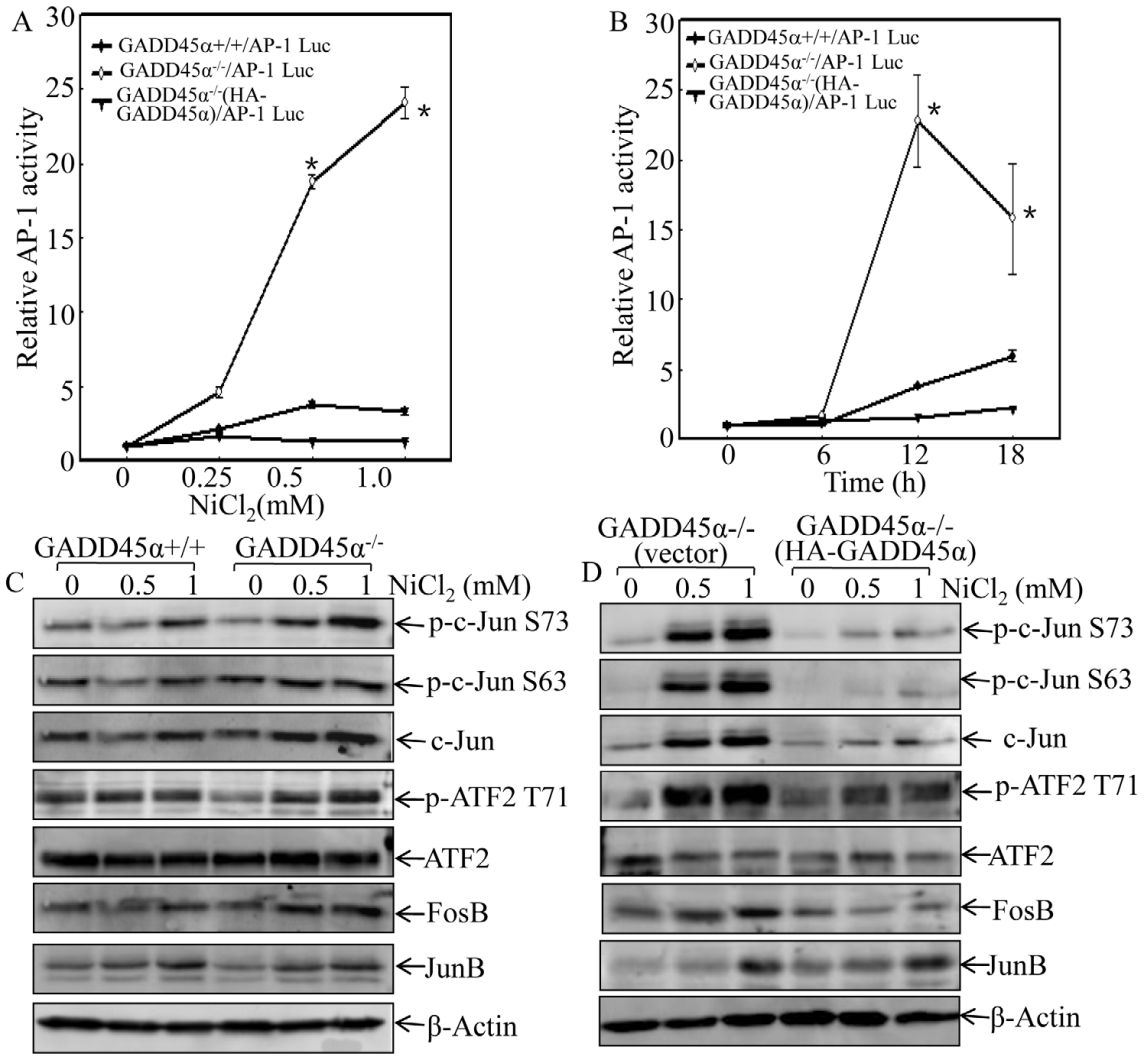
GADD45α inhibited AP-1 activation in nickel response. (A and B), AP-1 Luciferase reporter construct was stably transfected into GADD45α+/+, GADD45α−/− and GADD45α−/− (HA-GADD45α) cells. The stable transfectants as indicated in figures were exposed to NiCl_2_ at indicated dose for 12 hrs (A), or at 1 mM for indicated time periods (B). The cells were then extracted with luciferase assay buffer and luciferase activity was determined as described in our previous studies [30]. The results were presented in luciferase activity relative to medium control (Relative AP-1 activity). The asterisk (*) indicates a significant increase in comparison to GADD45α+/+ or GADD45α−/− (HA-GADD45α) cells (p<0.05). (C and D), the phosphorylation and expression of AP-1 components upon nickel exposure were evaluated in GADD45α+/+ and GADD45α−/− cell (C), or GADD45α−/− (vector) and GADD45α−/− (HA-GADD45α) cells (D) by western blot.

AP-1 is a main JNK/p38 downstream targeted transcription factor that plays an important role in the mediation of oxidative stress responses [31]. Thus, the effects of GADD45α protein expression on an AP-1-dependent transcription activity and potential AP-1 components involved in this regulation were further investigated. AP-1-luciferase reporter was transfected to GADD45α+/+, GADD45α−/− and GADD45α−/− (HA-GADD45α) cells, respectively. The transfectants were exposed to nickel and AP-1 activity was determined with luciferase activity assay. As shown in Fig. 2, nickel-induced AP-1 activity was much higher in GADD45α−/−/AP-1-Luc cells in comparison to that in GADD45α+/+ or GADD45α−/−(HA-GADD45α) cells in various doses and time points tested (Figs. 2A and 2B). Our results further indicated that GADD45α expression inhibited c-Jun phosphorylation/expression, ATF2 phosphorylation and Fos B expression following nickel exposure, while it did not affect Jun B protein expression (Figs. 2C and 2D). These data reveal that GADD45α inhibits the activation of JNK/p38 and their downstream transcription factor AP-1 upon nickel exposure.

### GADD45α Inhibited JNK/p38 Activation through Down-regulation of their Upstream Pathway

**Figure 3:**
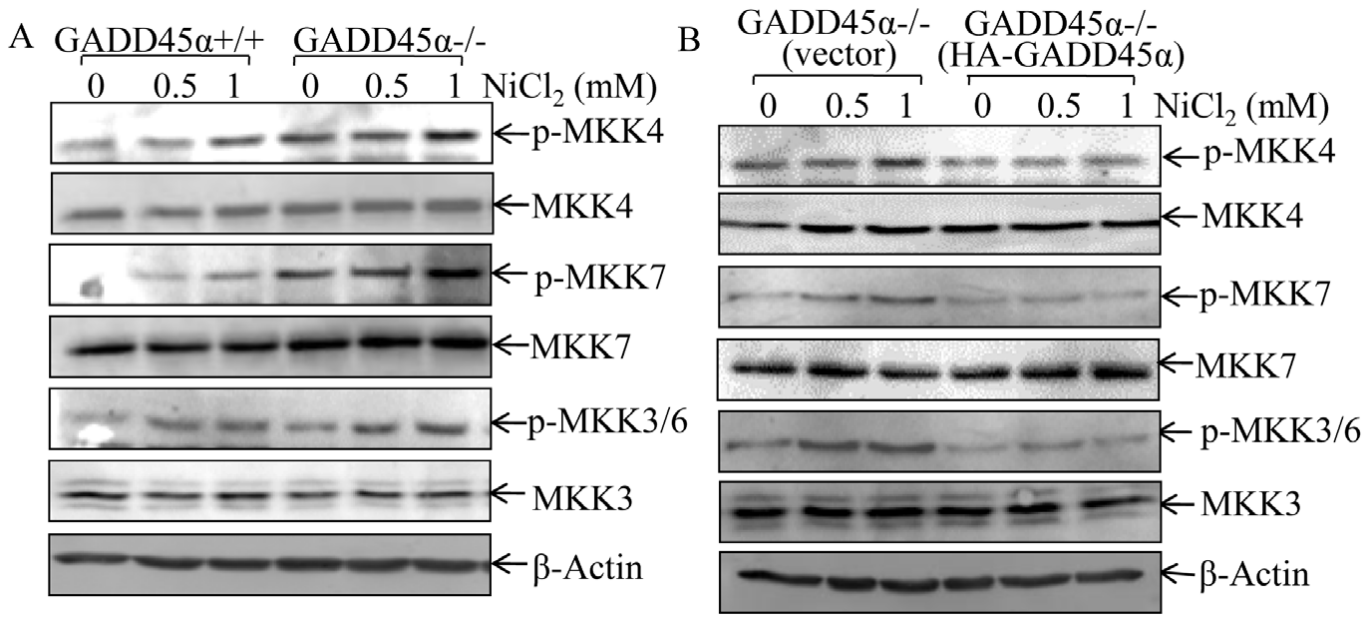
GADD45α inhibited MKKs activation following nickel exposure. (A and B), The indicated cells and their transfectants were exposed to nickel at various concentrations for 6 hrs, and activation of MKK4/7 and MKK3/6 were determined by western blot.

It has been well characterized that MKK4 and MKK7 are two of the upstream kinases that are directly responsible for phosphorylating JNK and the resultant in activation of JNK [32], while MKK3 and MKK6 are p38 upstream kinases, and phosphorylate p38 at Thr180 and Tyr182 and subsequently leading to p38 kinase activation [33]. Thus, we evaluated the effect of GADD45α on JNK/p38 upstream kinases activation upon nickel exposure by evaluating the MKK activation in GADD45α+/+, GADD45α−/− and GADD45α−/−(HA-GADD45α) cells. Consistent with the results demonstrated for JNK/p38 activation, nickel-induced activation of MKK4/7 and MKK3/6 in GADD45α−/− cells was much higher than those observed in GADD45α+/+ cells (Fig. 3A). This up-regulation of MKK4/7 and MKK3/6 in GADD45α−/− MEFs was completely impaired by the introduction of HA-GADD45α into GADD45α−/− cells (Fig. 3B). Moreover, there was no observable difference in the activation of MEK1/2, the upstream kinase for activating Erk, among the three types of cells (data not shown).

### Ectopic Expression of HA-GADD45α Inhibited MKKs -JNK/p38 Activation in HCT116 Cells

**Figure 4:**
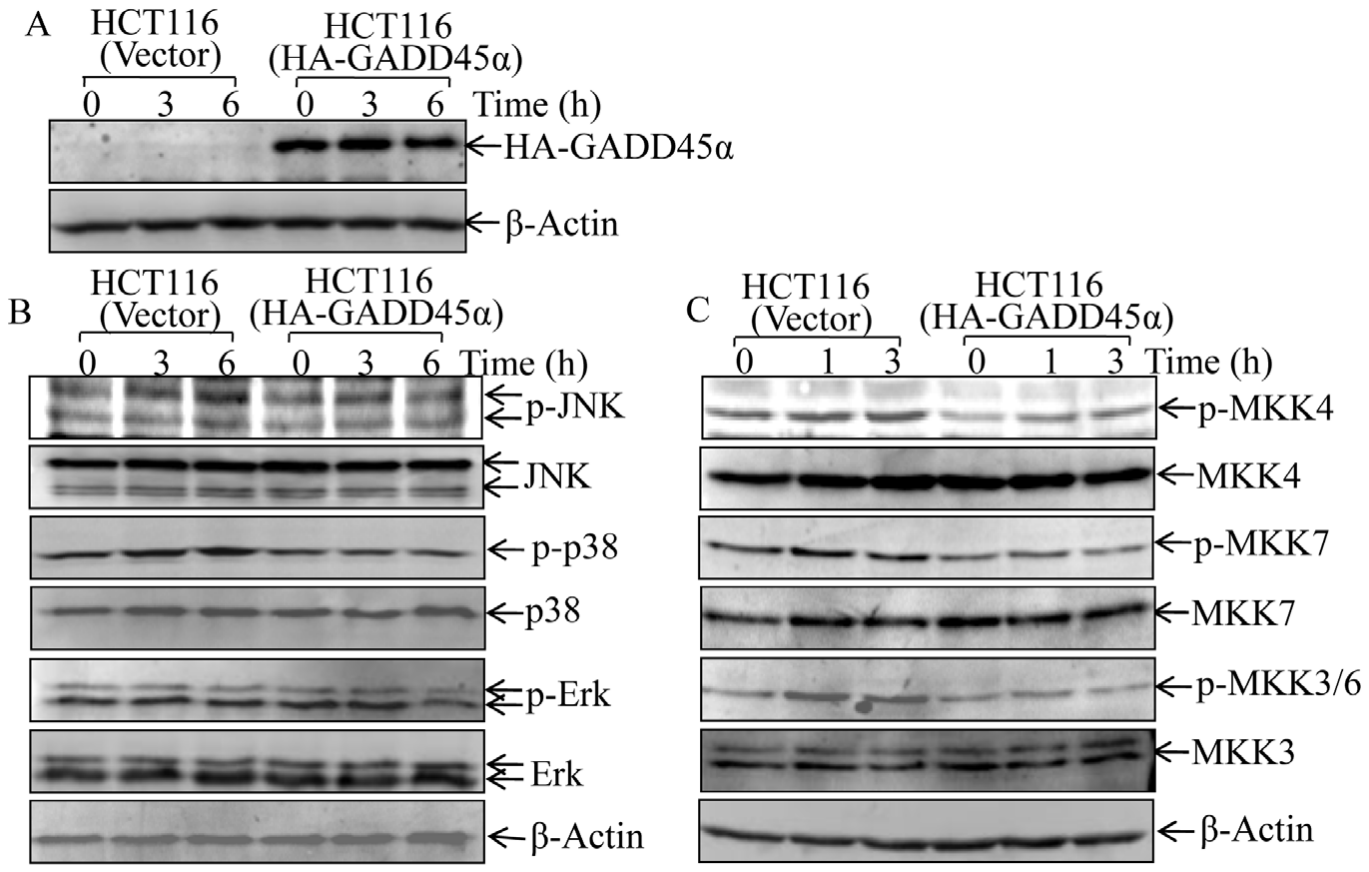
Ectopic expression of GADD45α inhibited MKK-JNK/p38 activation by nickel exposure in HCT116 cells. (A), HA-GADD45α expression construct was transfected into HCT116 cells, and stable transfectants, including HCT116 (vector) and HCT116 (HA-GADD45α), were established and identified. (B and C), The stable transfectants were exposed to NiCl_2_ (1 mM) for time periods as indicated. The total and phosphorylation protein expression of MKK4/7, MKK3/6, and JNK, as well as p38 were determined by western blot.

To clarify whether GADD45α inhibition of MKK-JNKs/p38 phosphorylation was cell-type specific, HA-GADD45α construct was stably transfected into HCT116 cells, and stable transfectant, HCT116 (HA-GADD45α) cells, was identified as shown in Fig. 4A. Not like MEF cells, the basal level of exogenous HA-GADD45α was equal to that with nickel treatment, which may be due to the mechanism that protein degradation mediated by ubiquitin-proteasome system was suppressed in colon cancer HCT116 cells [34]. HA-GADD45α overexpression in HCT116 cells specifically inhibited nickel-induced the activation of JNK and p38, but not Erk, in comparison with those in parental HCT116 cells with vector control transfectant, HCT116 (vector) (Fig. 4B). Again, HA-GADD45α overexpression also impaired the activation of MKK4/7 and MKK3/6 upon nickel exposure (Fig. 4C). These results indicate that the down-regulation of MKK-JNK/p38 pathway by GADD45α is not cell-type specific.

### GADD45α Inhibited MKK-JNK/p38 Pathway by Upregulation of PP2Cα Expression

**Figure 5:**
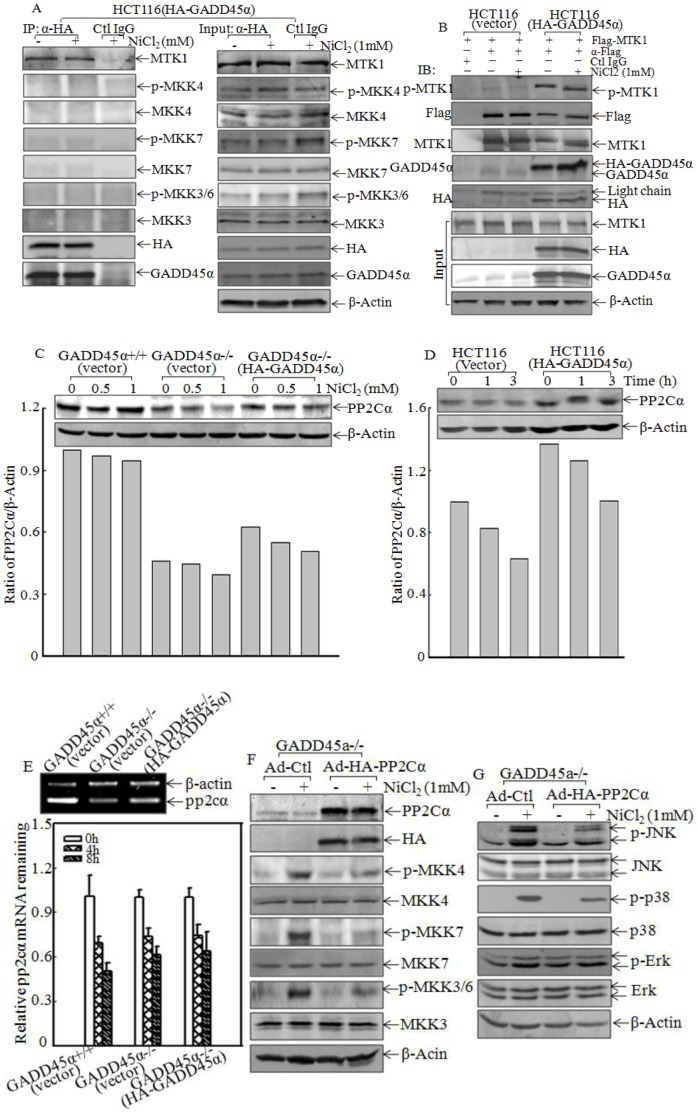
GADD45α regulated PP2Cα expression. (A), HCT116 (HA-GADD45α) were treated with NiCl_2_ (1 mM), and cells were extracted for immunoprecipitation using anti-HA antibody. Input and immunoprecipitates were subjected to western blot and probed with specific antibodies as indicated. (B), HCT116 (vector) and HCT116 (HA-GADD45α) stable transfectants were transiently transfected with Flag-MTK1 expression constructs. The transfectants were treated by 1 mM NiCl_2_, and extracted for immunoprecipitation using anti-Flag antibody. Phosphor-MTK1 Thr1493, MTK1, Flag, HA and GADD45α were determined by western blot using specific antibodies. (C and D), PP2Cα was evaluated in GADD45α+/+ (vector) and GADD45α−/− (HA-GADD45α) cells after 6 hrs treatment with NiCl_2_ or in HCT116 (vector) and HCT116 (HA-GADD45α) cells exposed to 1 mM NiCl_2_. The ratio of PP2Cα/β-Actin was determined using the software of ImageQuant 5.2 (GE healthcare, Pittsburgh, PA, USA). The asterisk (*) demonstrates a significant difference between the indicated cells (p<0.05). (E), *pp2cα* mRNA in GADD45α+/+ (vector), GADD45α−/− (vector) and GADD45α−/− (HA-GADD45α) cells were measured by RT-PCR (top panel) or *pp2cα* mRNA stability after treated by 5 µM Act D as indicated time was determined by real time PCR (bottom panel). (F and G), total and phosphorylation of MKKs and MAPKs were detected in GADD45α−/− (Ad-control) and GADD45α−/− (Ad-HA-PP2Cα) as indicated.

Although three members of GADD45 protein, including α, β and γ, could bind to MEKK4/MTK1 [26], only GADD45β has been shown to inhibit JNK activity through binding to MKK7 directly [35]. To elucidate the molecular mechanisms underlying the GADD45α inhibition of MKK-JNK/p38 kinase activation in nickel response, we treated HCT116 (HA-GADD45α) transfectant with nickel and the cell extracts were used for co-immunoprecipitation using anti-HA antibody. As shown in Fig. 5A, there was a strong MTK1 band presented in the co-immunoprecipitation complexes, whereas all other kinases, such as MKK4, MKK7 and MKK3/6, were not detectable, indicating that GADD45α did not show any binding activity to MAPKKs (MKK4/7 and MKK3/6), which have been reported in previous studies [35]. In contrast, all those proteins were detectable in input of whole-cell extracts (Fig. 5A). These results suggest that GADD45α specifically binds to MTK1. Since MTK1 dimerization and auto-phosphorylation is the critical step for its activation [26], we determined whether GADD45α could interact with and interrupts MTK1 auto-phosphorylation. We transiently transfected Flag-MTK1 into HCT116 (vector) and HCT116 (HA-GADD45α) cells, and immunoprecipitation was performed using α-Flag antibody. The results showed that although expression level of Flag-MTK1 in the immune-pull down complexes from HCT116 (vector) was much higher than that in HCT116 (HA-GADD45α) cells, ectopic expression of GADD45α in HCT116 (HA-GADD45α) cells markedly increased MTK1 phosphorylation at Thr 1493 (p-MTK1) in comparison to those in HCT116 (vector) cells (Fig. 5B). These results suggest that GADD45α enhances the MTK1 activation through promoting its auto-phosphorylation at Thr 1493. It was not consistent with our findings that MTK1 downstream kinases MKK4/7 and MKK3/6 were repressed in present of GADD45α following nickel exposure (Figs. 3A and 3B) and further revealed that GADD45α might act on the phosphotase of MKK4/7 and MKK3/6. PP2Cα have been identified as a phosphotase responsible for dephosphorylation of MKK4/7 and MKK3/6 [36], [37]. We therefore compared PP2Cα protein levels between GADD45α+/+ and GADD45α−/− cells. The results showed that PP2Cα protein level in GADD45α−/− cells was dramatically reduced in compared to that in GADD45α+/+ cells, although nickel exposure did not show observable decreases of PP2Cα expression (Fig. 5C). This finding was further verified in HCT116 cell with ectopic expression of HA-GADD45α (Fig. 5D). To determine whether GADD45α regulates PP2Cα upregulation at mRNA level, RT-PCR was performed to measure mRNA levels of *pp2cα* among GADD45α+/+ (vector), GADD45α−/− (vector) and GADD45α−/− (HA-GADD45α) cells. The results showed that deletion of GADD45α attenuated *pp2cα* mRNA expression (Fig. 5E, top panel), whereas *pp2cα* mRNA stability was comparable among these three cells (Fig. 5E, bottom panel). These results suggest that GADD45α may play an important role in regulating *pp2cα* gene transcription. To further evaluate the role of PP2Cα in GADD45α-mediated downregulation of MKK-JNK/p38 activation, we used Ad-HA-PP2Cα to constitutional express HA-PP2Cα in GADD45α−/− cells. As shown in Figs. 5F and 5G, constitutional expression of HA-PP2Cα attenuated MKK3/6 and MKK4/7, as well as their downstream JNK and p38 activation following nickel exposure. These results demonstrate that GADD45α inhibits MKK-JNK/p38 activation through promoting PP2Cα expression in nickel response.

## Discussion

The IARC classifies nickel compounds as group 1 carcinogens (confirmed carcinogen) for human [38]. It has been thought that non-genotoxic effect of nickel compounds is responsible for their carcinogenic activity [39]. In the present study, we found that nickel treatment induced GADD45α expression. Unlike our previous reports showing GADD45α up-regulation of JNK pathway upon arsenite exposure [19], the induced GADD45α expression by nickel exhibited an inhibitory effect on MKK-JNK/p38/AP-1 pathway. Our subsequent study demonstrated that the inhibitory effect of GADD45α on MKK-JNK/p38/AP-1 pathway was mediated through up-regulation of PP2Cα expression. Our study identified a novel function of GADD45α as a negative regulator for stress responses, including MKK-JNK and MKK-p38 activation.

The JNK is implicated in several physiological processes, including proliferation, apoptosis and differentiation. For example, JNK signaling pathway activation has been implicated in the apoptosis induction in different cell types [40]. The most convincing evidence comes from the observation that the JNK1 and JNK2 double knockout cells are resistant to the apoptosis induced by UV irradiation [40]. Our previous report also demonstrates that arsenite is able to induce sustained JNK activation and cellular apoptosis, and more importantly, the expression of dominant-negative JNK1 in Cl41 cells abrogates the apoptotic response [41]. Our further studies show that the coordination of JNK1 and JNK2 is required for the apoptotic responses caused by arsenite in MEFs because knockout of either JNK1 or JNK2 totally blocks arsenite-induced DNA fragmentation, caspase-3 activation and PARP cleavage [27], and such arsenite-induced cell apoptosis is mediated through IKK/p50/GADD45α/MKK4/JNK axis [19]. In contrast to apoptotic effect of JNK, there is accumulated evidence supporting that JNK plays a role in cancer development. Numerous tumor cell lines have been reported to possess constitutively active JNK [42]. The expression of JNK1 was increased markedly in breast cancer tissue compared to normal samples [43]. JNK1 has been reported to play a pivotal role in the expression of the key signature genes and the prognostic outcomes of human hepatocellular carcinoma [44]. Recently reports indicate that p38 can directly phosphorylate C-terminus of GSK3β, which inhibits the ubiquitination and degradation of β-catenin. This β-catenin accumulation results in promoting cell survival in specific tissues [45]. Nickel, an environmental and occupational carcinogen, is associated with an increase in high risk for lung cancer and nasal cancer [2]. Our previous studies demonstrate that JNK1 is responsible for the nickel-related COX-2 induction, a key molecule involved in inflammatory response and tumor development [46]. Nickel exposure induces the activation of NFκB, and subsequently leads to COX-2 induction, and protects nickel-exposed BEAS-2B cells from apoptosis [47]. Nickel exposure also leads to NFAT activation and TNF-α expression in BEAS-2B cells [48], and that TNF-α treatment can result in COX-2 expression, which is responsible for TNF-α-mediated cell transformation through NFAT-dependent pathway. Our most recent study discloses a novel function of JNK1 in the modulation of HIF-1α stabilization upon nickel exposure through regulation of Hsp90/Hsp70 expression as well as HDAC6-mediated Hsp90 acetylation modification [3], whereas JNK2 activation plays an essential role in regulation of hif-1α mRNA stability [49]. Although the molecular mechanisms underlying JNK-mediated apoptotic effect of arsenite and oncogenic effect of nickel treatment are not understood yet, we have noted the differential effects of GADD45α on JNK activation following arsenite and nickel stimulation. We found that GADD45α induction upon arsenite treatment is crucial for JNK activation [3], [19], [27], whereas GADD45α induction by nickel exposure provides an inhibitory effects on JNK and p38 activation as demonstrated in our current study. Since our results demonstrate a key role of HIF-1α induction in nickel-induced cell transformation [3], we anticipate that GADD45α induction by nickel plays a role in nickel-induced carcinogenic effect. We also anticipate that different biological effects of GADD45α expression between nickel and arsenite exposure may account for distinct final outcomes of two exposures in various experimental systems. In addition, our studies indicate that arsenite-induced GADD45α expression is mainly through inhibition of GADD45α protein degradation and to less extent at transcription level [19], whereas nickel-induced GADD45α expression is through modulating mRNA expression level (Fig. 1B). The significance of the differential levels of GADD45α expression regulation and the molecular mechanisms underlying this difference between arsenite and nickel exposures will be one of interesting area needed to be further explored in our future studies.

GADD45α is considered as a cancer susceptibility gene, and GADD45α overexpression is connected with pancreatic cancer development [50]. Most previous studies indicate that GADD45α mediates cell apoptosis through activating JNK and/or p38 pathways [18], [51], whereas GADD45β or GADD45γ can bind to N-terminus of MTK1/MEKK4 and interrupt its interaction with C-terminus, the release of kinase domain in C-terminus, which leads to MTK1/MEKK4 homodimerization, auto-phosphorylation and activation in normal culture cells without oxidative stress conditions [20]. Activated MTK1/MEKK4 subsequently mediates phosphorylation of MKK4/7 [52] and their downstream JNK activation [32], as well as MKK3/6 [52] and their downstream p38 activation [21]. Consistently, we demonstrate that GADD45α induction is crucial for JNK activation through enhancing MKK4 phosphorylation upon arsenite treatment [19]. Contradictiously, GADD45β has been shown to bind to MKK7 and inhibit JNK activation [35]. Our current study indicates that GADD45α only binds to MTK1/MEKK4 and enhances its auto-phosphorylation at Thr1493, whereas there was no observable protein-protein interaction between GADD45α and MKKs. These data suggest that GADD45α inhibitory effect on MKK-JNK/p38 activation might be via upregulation of phosphatase of MKK-JNK/p38, rather than down-regulating upstream kinase. Our further elucidation demonstrates that GADD45α deletion attenuates PP2Cα expression, which is known as a phosphatase of MKK4/7 and MKK3/6 [36], [37]. Moreover, we found that GADD45α is essential for mediation of pp2cα mRNA expression and that constitutional expression of GADD45α in GADD45α−/− cells brought PP2Cα expression back to normal level in GADD45α+/+ cells. Collectively, we conclude that GADD45α induction by nickel exposure provides an inhibitory effect on MKK-JNK/p38 pathways via promotion of pp2cα mRNA expression.

In conclusion, our present study, for the first time to best of our knowledge, demonstrates a novel effect of GADD45α on inhibiting JNK and p38 activity through promoting PP2Cα expression, and subsequently leading to inhibition of MKK-JNK/p38 activation and their downstream transcriptional factor AP-1 transactivation in cellular response to nickel exposure. Such GADD45α induction prevents sustained JNK activation and JNK-mediated cellular apoptotic response following nickel exposure. Considering an important role of JNK activation in mediation of HIF-1α expression and cell transformation following nickel exposure [3], we anticipate that this new function of GADD45α is associated with nickel carcinogenic effect. This novel information will provide insight into understanding the big picture of GADD45α in the regulation of cellular responses to oxidative stress and environmental carcinogens.
